# Discussion of tumor mutation burden as an indicator to predict efficacy of immune checkpoint inhibitors: A case report

**DOI:** 10.3389/fonc.2022.939022

**Published:** 2022-08-03

**Authors:** Mingrui Wu, Lan Liang, Xiaotian Dai

**Affiliations:** ^1^ Department of Respiratory and Critical Care Medicine, Affiliated People‘s Hospital of Chongqing Three Gorges Medical College, Chongqing, China; ^2^ Department of Respiratory and Critical Care Medicine, The First Affiliated Hospital of Army Medical University, Chongqing, China

**Keywords:** immune checkpoint inhibitors, prognostic biomarker, treatment efficacy, tumor mutation burden (TMB), immunotherapy

## Abstract

There are many treatment options for advanced lung cancer, among which immunotherapy has developed rapidly and benefited a lot of patients. However, immunotherapy can only benefit a subgroup of patients, and how to select patients suitable for this therapy is critical. Tumor mutation burden (TMB) is one of the important reference indicators for immune checkpoint inhibitors (ICIs). However, there are many factors influencing the usage of this indicator, which will lead to considerable consequences if not treated well. In this study, we performed a case study on a male advanced lung squamous cell carcinoma patient of age 83. The patient suffered from “cough and sputum”, and did chest *CT* scans on 24 October 2018, which showed “a mass-like mass in the anterior segment of the right lung upper lobe, about 38mm×28mm”. He was treated with systemic chemotherapy; however, the tumor was still under progression. Although PD-L1 was not tested in gene testing, he had a TMB value of 10.26 mutations/Mb with a quantile value 88.63%. Thus, “toripalimab injection” was added as immunotherapy and the size of the lesion decreased. In summary, we adopted a clinical case as the basis to explore the value and significance of TMB in immunotherapy in this study. We hope that more predictive molecular markers will be discovered, which will bring more treatment methods for advanced lung cancer.

## Introduction

Lung cancer, also known as lung carcinoma, is one of the most prevalent malignant tumors worldwide (1, 2). Patients with lung cancer suffer frequently from recurrence and metastasis, while a few patients even cannot determine the original lesion (1, 3–5). Advanced lung cancer patients have diversified treatments. With the advancement of precision medicine and further exploration at the molecular level, molecular targeted drugs are widely used. It brings new treatment methods to lung cancer patients in addition to surgery and chemoradiotherapy. Targeted drug resistance cannot be avoided (6–11), and therefore, it is vital to actively find new treatments to prolong the survival time of patients.

In recent years, immune checkpoint inhibitors (ICIs) have developed rapidly, and monoclonal antibodies CTLA-4, PD-1, and PD-L1 have greatly extended the survival time of advanced lung cancer patients (12, 13). However, the efficacies of ICIs depend on the genetics of specific patients. Thus, it is critical to identify indicators that could predict the performance of different ICIs on a patient. Widely used indicators for immunotherapy include programmed death ligand-1 (PD-L1), mismatch repair (MMR), microsatellite instability (MSI), and tumor mutation burden (TMB). TMB is defined as the total number of substitutions and insertions, deletion mutations per megabase in the exon-coding region of a tumor specimen. High TMB usually means more neoantigens, and thus, immune system will have more chance to identify tumor cells. However, since TMB is an exome-wide indicator, it is not specific for detecting one specific mutation and one ICI. Thus, sometimes the patients selected based on this criterion will not benefit from immunotherapy. As a result, there is still a debate on the usage of TMB, and it is critical to show some clinical cases with immunotherapy and high TMB. This kind of cases can further guide the appropriate usage of this important indicator and help to select beneficiaries of immunotherapy in advance. In this paper, we use one advanced lung squamous cell carcinoma case as a discussion of the significance of TMB.

## Case study

**Figure 1:**
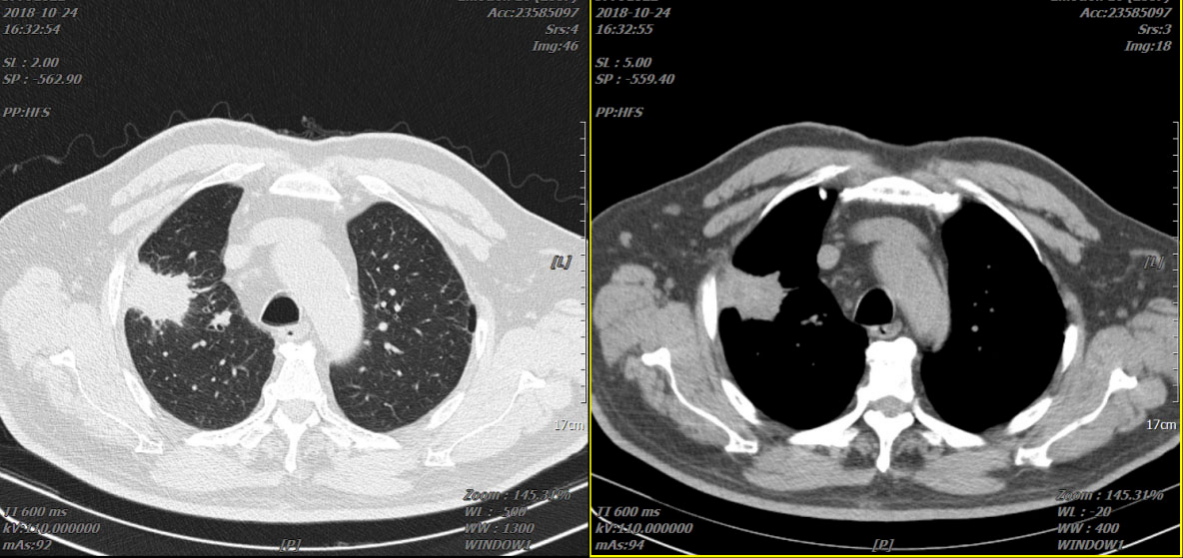
Two representative CTs of the patient on 24 October 2018.

The patient suffered from “cough and sputum”, and he underwent chest *CT* scans on 24 October 2018 which showed “a mass-like mass in the anterior segment of the right lung upper lobe, about 38mm×28mm” (**Figure 1**).

Lung biopsy: (the right upper lung) Squamous cell carcinoma infiltration is seen in the large amount of fibrous collagen tissue. Whole-body PET/CT: 1. Peripheral lung cancer of the anterior segment of the right lung upper lobe involves the adjacent pleura; 2. Multiple metastases in the right pleura. Diagnosis is “right upper lung squamous cell carcinoma (T3N0M1a stage IVA)”. Ten-gene detection of puncture tissue: 10-gene mutations such as EGFR were not detected.

**Figure 2:**
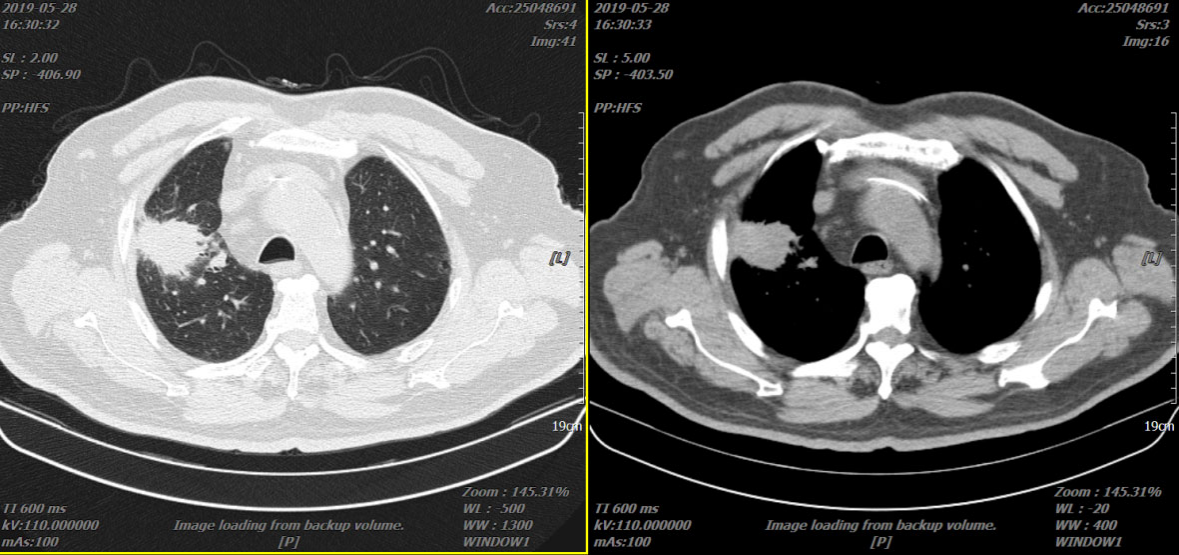
Two representative CTs of the patient on 28 May 2019.

The patient was given “paclitaxel liposome + nedaplatin”, “docetaxel + nedaplatin”, and “gemcitabine + nedaplatin” systemic chemotherapy on 28 November 2018. During the reexamination of the lung lesions, there was no significant reduction compared with the baseline. Reexamination of the chest *CT* scan on 28 May 2019 revealed that the lung lesions were slightly enlarged, but less than 20% (**Figure 2**). The patient refused to continue chemotherapy. Then the patient was given “recombinant human endostatin injection” for antitumor angiogenesis and “afatinib” second-line systemic therapy.

**Figure 3:**
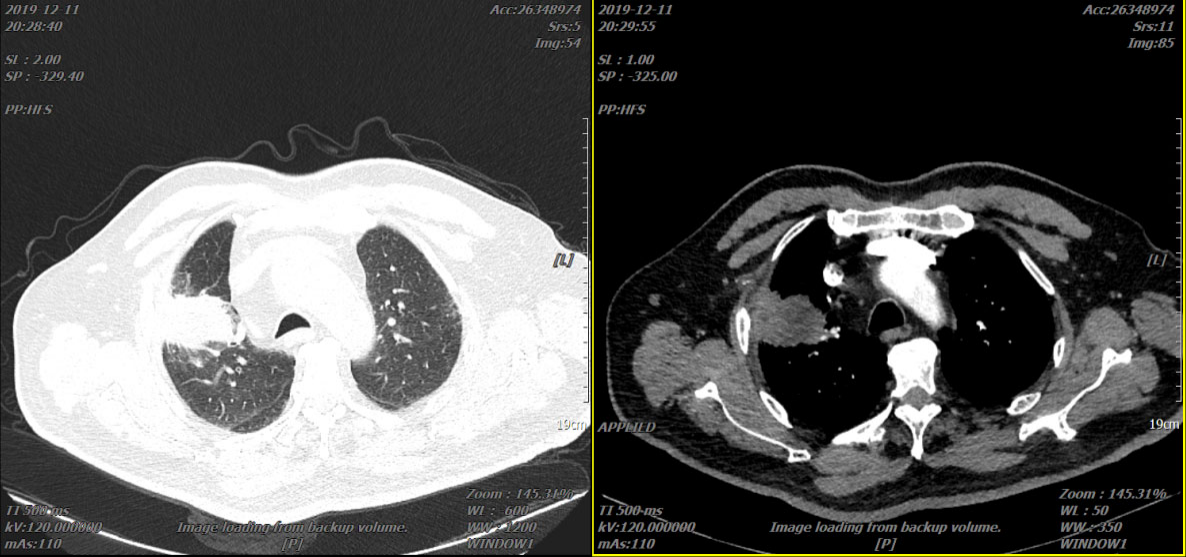
Two representative CTs of the patient in 21 December 2019.

Reexamination of the chest *CT* scan on 21 December 2019 revealed that the lung lesions were significantly enlarged (49 mm × 34 mm) (**Figure 3**), and bone imaging revealed new bone metastasis. The progress of the disease was evaluated. The blood gene test did not find the mutant gene. PD-L1 was not tested, but TMB had 10.26 mutations/Mb, and the quantile value was 88.63%.

**Figure 4:**
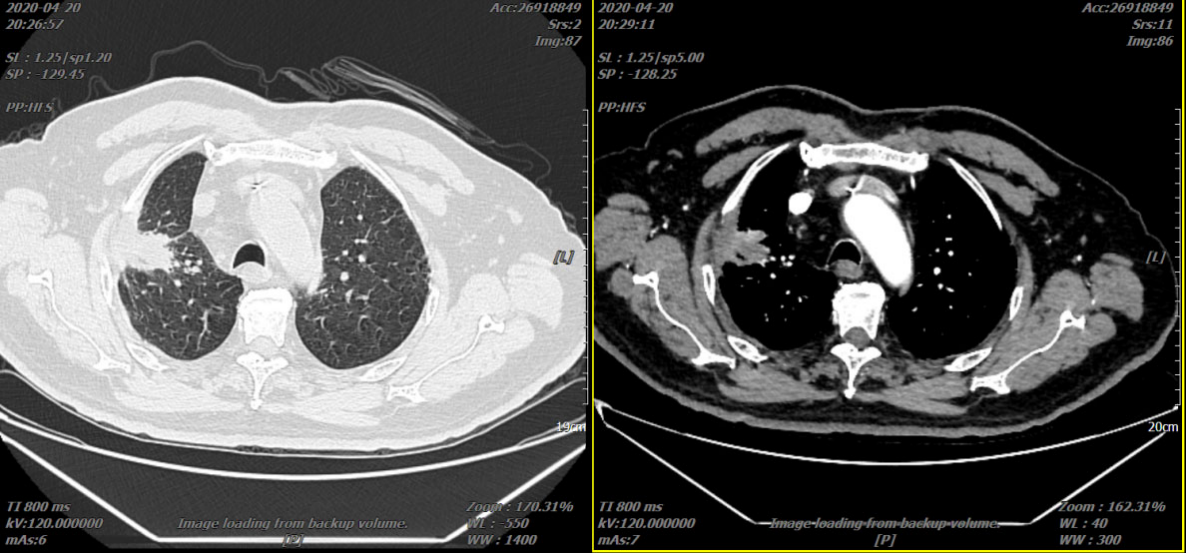
Two representative CTs of the patient on 20 April 2020.

On 14 January 2020, the systemic chemotherapy of “paclitaxel liposome + nedaplatin” was given again, the “recombinant human endostatin injection” antitumor angiogenesis treatment was continued, and the “toripalimab injection” was added with immunotherapy and continuous follow-up. On 20 April 2020, reexamination of the chest *CT* scan showed that the right lung lesion shrank to 33 mm × 30 mm (**Figure 4**).

**Figure 5:**
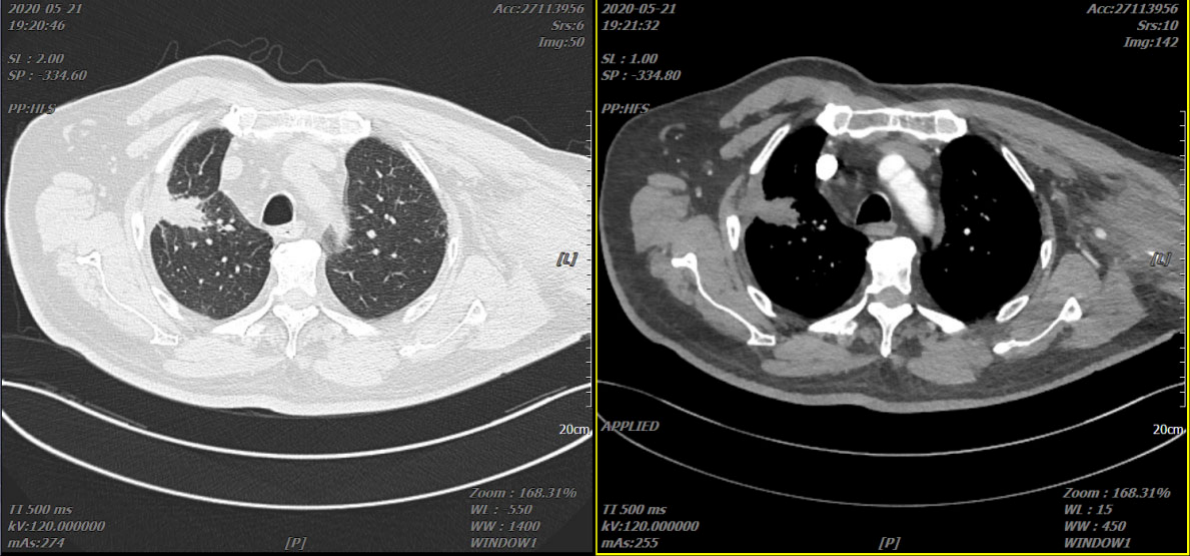
Two representative CTs of the patient on 21 May 2020.

Reexamination of the chest *CT* scan on 21 May 2020 showed that the right lung lesion shrank to 31 mm × 21 mm (**Figure 5**). Due to multicyclic chemotherapy, the patient began to show bone marrow suppression. On 11 August 2020, the number of circulating tumor cells (CTCs) was detected to be 2. Chemotherapy was stopped, and the “recombinant human endostatin injection” combined with “toripalimab injection” was continued.

**Figure 6:**
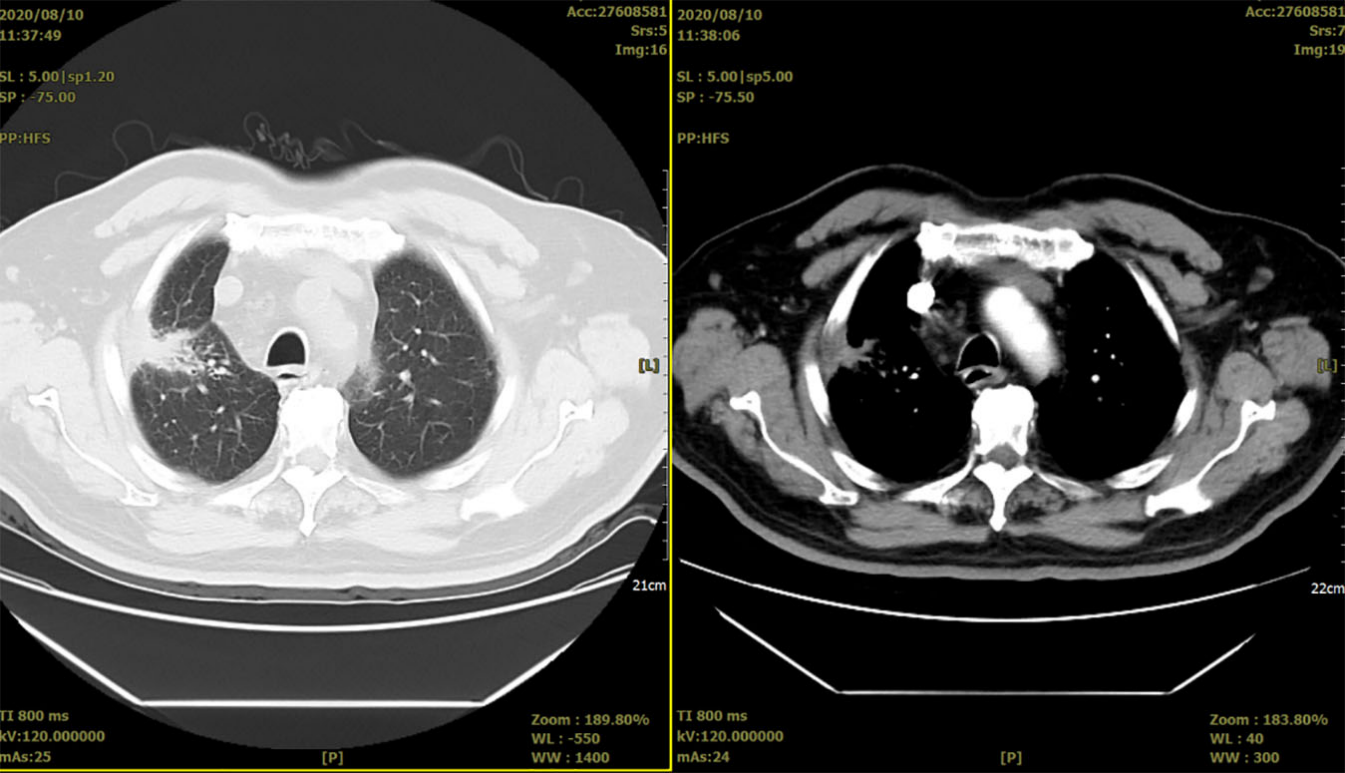
Two representative CTs of the patient on 10 August 2020.

On 10 August 2020, reexamination of the chest *CT* scan revealed that the lung lesions have not changed much from the previous one (**Figure 6**).

## Discussion

It is critical to identify prognostic biomarkers that can predict the efficacy of a treatment, as well as the recurrence and survival of cancer patients. Many studies have focused on identifying these biomarkers (14–18), among which TMB received more and more attention. TMB refers to the sum of substitution, insertion, and deletion mutations in the coding region of the evaluated tumor cell genes (19). We can also simply think of how many cell genes in the tumor tissue have mutated. If tumor cells have more gene mutations (i.e., high TMB), they are more likely to produce abnormal proteins; these proteins are called neoantigens. Studies have shown that every 150 non-synonymous mutations produce one to two neoantigens, and these antigens can be the immune system that sees through and activates the body’s T-cell immune response (20–24). TMB is a pan-cancer immunotherapy biomarker, which has proven to be useful in almost all cancers including lung cancer, colorectal cancer, melanoma, endometrial cancer, cervical cancer, and bladder cancer.

Previous studies often used PD-L1 as a molecular marker for screening immune expression benefits (25). However, it was found in the phase III clinical trial of CheckMate 026 that even if the PD-L1 expression level is greater than 50%, patients cannot fully benefit from nivolumab treatment. In another study which based on this result, in the subgroup with high TMB, nivolumab had an ORR of 47%, while chemotherapy combined with nivolumab was 28%, and PFS was 9.7 months for the former and 5.8 months for the latter. It shows that nivolumab is significantly better than chemotherapy (26). From a genetic point of view, the more non-synonymous the mutations, the more the neoantigens may be recognized by the autoimmune system, and the final immunotherapy effect will be stronger. Therefore, the higher the TMB, the more it benefited from ICIs (27, 28). Both CheckMate 568 phase II and CheckMate 227 phase III studies have also confirmed that regardless of the level of PD-L1 expression, nivolumab combined with ipilimumab has benefited populations. However, the use of TMB as a molecular marker to predict efficacy can only be used in patients with TMB ≥10 mut/Mb; it was found that PFS was longer than that in the chemotherapy group, and the ORR was also higher. Therefore, we suggest that TMB ≥10 mut/Mb should often be considered as an effective cutoff index for screening patients who benefit from immunotherapy (29, 30).

A number of studies have shown that lung cancer patients with high TMB have better efficacy and prognosis after they used ICIs. In 2018, the National Comprehensive Cancer Network even included TMB in its lung cancer guidelines. Yarchoan et al. published an article in the New England Journal in 2017. The study compared the ORR value of TMB in the treatment of 27 kinds of tumors with PD-1 or PD-L1. The results showed that TMB was related to the ORR value of 55% of patients, and the efficacy prediction of lung cancer is more accurate. The linear relationship formula: overall effective rate = 10.8×log(X)-0.7, where “X” represents the number of somatic DNA mutation load per megabase (21). In the CheckMate 026 study, nivolumab- and platinum-containing chemotherapy was used in the first-line treatment of advanced lung cancer. WES was used to measure TMB, and the patients were divided into three groups (<100 mutations, 100–242 mutations, ≥243 mutations); the results showed that the high TMB subgroup (≥243 mutations) patients treated with nivolumab had higher ORR and longer PFS than the chemotherapy group (31). In February 2018, Annals of Oncology published two studies of CheckMate 017 and CheckMate 057. After 3-year minimum follow-up, whether it is squamous or non-squamous NSCLC, the OS value of the nivolumab group was significantly prolonged than that of the docetaxel group (HR = 0.70, 95% CI: 0.61–0.81), and they got benefits from continued survival (32). In addition, an article published on Nat Genet in 2019 (16) brought light to the prediction of the efficacy of TMB as ICIs. This is the largest clinical study on both of them so far. It includes 1,662 patients who have received ICIS treatment and also covers 10 types of malignant tumors. The study found that in most patients, the overall survival rate of 20% patients with TMB in the high is higher than that in 80% patients with TMB in the low, which confirms that TMB can be used as a biomarker for predicting the efficacy of ICIs. It has contributed to exploring the threshold of TMB.

The above paragraphs mentioned the use of WES to measure the TMB value, but the current clinical testing is mainly based on major genetic companies’ Panel, which may be closely related to the low price. It causes many uncertain factors, such as different test reagents, or different numbers of genes, and different set standard parameter values. However, regardless of the TMB detection method, the level of TMB expression needs to be considered related to many factors. Chalmers et al. (33) have found that there is a significant correlation between TMB expression and age. The study showed that median TMB at age 10 was 1.67 mutations/Mb, and median TMB at age 88 was 4.50 mutations/Mb. At the same time, the study also predicted the TMB of between age 10 and age 90 according to A linear model, and the final difference was 2.4-fold. Rizvi et al. (34) also studied the efficacy of pembrolizumab in the treatment of advanced lung cancer and found that it is closely related to smoking, changes in DNA repair pathways, and high expression of neoantigens in tumor tissues. These factors are also related to high TMB.

To sum up, immunotherapy has brought all cancer treatments into another era of precision medical care, with unlimited potential. The current research on its efficacy is generally considerable, and most patients can benefit from it. However, research on indicators for predicting its efficacy is still at the tip of the iceberg, and there is no clear single molecular marker that can determine the efficacy of ICIs. Therefore, it is hoped that more studies that can effectively predict molecular markers are in full swing. At present, it is certain that the combination of immunotherapy and existing treatment methods will be a key subject of lung cancer treatment research. At the same time, we are also looking forward to more new methods, but how to optimize the allocation of these combinations has a long way to go.

## Data availability statement

The original contributions presented in the study are included in the article/supplementary material. Further inquiries can be directed to the corresponding author.

## Author contributions

LL and MW was responsible for study conception and design; authors MW and XD were responsible for data collection and analysis; author LL were responsible for drafting the manuscript. All authors contributed to manuscript revision, read, and approved the submitted version.

## Conflict of interest

The authors declare that the research was conducted in the absence of any commercial or financial relationships that could be construed as a potential conflict of interest.

## Publisher’s note

All claims expressed in this article are solely those of the authors and do not necessarily represent those of their affiliated organizations, or those of the publisher, the editors and the reviewers. Any product that may be evaluated in this article, or claim that may be made by its manufacturer, is not guaranteed or endorsed by the publisher.

## References

[1] Sung H Ferlay J Siegel RL Laversanne M Soerjomataram I Jemal A. Global cancer statistics 2020: globocan estimates of incidence and mortality worldwide for 36 cancers in 185 countries. CA Cancer J Clin (2021) 71:209–49. doi: 10.3322/caac.21660 33538338

[2] Cienfuegos-Jimenez O Vazquez-Garza E Rojas-Martinez A. CAR-NK cells for cancer therapy: molecular redesign of the innate antineoplastic response. Curr Gene Ther (2022) 22(4):303–18. doi: 10.2174/1566523222666211217091724 34923939

[3] Liu H Qiu C Wang B Bing P Tian G Zhang X. Evaluating dna methylation, gene expression, somatic mutation, and their combinations in inferring tumor tissue-of-origin. Front Cell Dev Biol (2021) 9:619330. doi: 10.3389/fcell.2021.619330 34012960 PMC8126648

[4] Wang F Yang J Lin H Li Q Ye Z Lu Q. Improved human age prediction by using gene expression profiles from multiple tissues. Front Genet (2020) 11:1025. doi: 10.3389/fgene.2020.01025 33101366 PMC7546819

[5] Tanaka E Uchida D Shiraha H Kato H Ohyama A Iwamuro M. Promising gene therapy using an adenovirus vector carrying reic/dkk-3 gene for the treatment of biliary cancer. Curr Gene Ther (2020) 20:64–70. doi: 10.2174/1566523220666200309125709 32148193

[6] Jia Y Yun CH Park E Ercan D Manuia M Juarez J. Overcoming EGFR(T790M) and EGFR(C797S) resistance with mutant-selective allosteric inhibitors. Nature (2016) 534:129–32. doi: 10.1038/nature17960 PMC4929832 27251290

[7] Biswas R Ghosh D Dutta B Halder U Goswami P Bandopadhyay R. Potential non-coding rnas from microorganisms and their therapeutic use in the treatment of different human cancers. Curr Gene Ther (2021) 21:207–15. doi: 10.2174/1566523220999201230204814 33390136

[8] Han X Kong Q Liu C Cheng L Han J. SubtypeDrug: a software package for prioritization of candidate cancer subtype-specific drugs. Bioinformatics (2021) 37(16):2491–3. doi: 10.1093/bioinformatics/btab011 33459772

[9] Di J Zheng B Kong Q Jiang Y Liu S Yang Y. Prioritization of candidate cancer drugs based on a drug functional similarity network constructed by integrating pathway activities and drug activities. Mol Oncol (2019) 13:2259–77. doi: 10.1002/1878-0261.12564 PMC6763777 31408580

[10] Liu C Wei D Xiang J Ren F Huang L Lang J. An improved anticancer drug-response prediction based on an ensemble method integrating matrix completion and ridge regression. Mol Ther Nucleic Acids (2020) 21:676–86. doi: 10.1016/j.omtn.2020.07.003 PMC7403773 32759058

[11] Liu X Yang J Zhang Y Fang Y Wang F Wang J. A systematic study on drug-response associated genes using baseline gene expressions of the cancer cell line encyclopedia. Sci Rep (2016) 6:22811. doi: 10.1038/srep22811 26960563 PMC4785360

[12] Nishino M Ramaiya NH Hatabu H Hodi FS. Monitoring immune-checkpoint blockade: response evaluation and biomarker development. Nat Rev Clin Oncol (2017) 14:655–68. doi: 10.1038/nrclinonc.2017.88 PMC5650537 28653677

[13] Meng Y Lu C Jin M Xu J Zeng X Yang J. A weighted bilinear neural collaborative filtering approach for drug repositioning. Brief Bioinform (2022) 23(2):bbab581. doi: 10.1093/bib/bbab581 35039838

[14] Yang M Yang H Ji L Hu X Tian G Wang B. A multi-omics machine learning framework in predicting the survival of colorectal cancer patients. Comput Biol Med (2022) 146:105516. doi: 10.1016/j.compbiomed.2022.105516 35468406

[15] Yang J Ju J Guo L Ji B Shi S Yang Z. Prediction of HER2-positive breast cancer recurrence and metastasis risk from histopathological images and clinical information *via* multimodal deep learning. Comput Struct Biotechnol J (2022) 20:333–42. doi: 10.1016/j.csbj.2021.12.028 PMC8733169 35035786

[16] Samstein RM Lee CH Shoushtari AN Hellmann MD Shen R Janjigian YY. Tumor mutational load predicts survival after immunotherapy across multiple cancer types. Nat Genet (2019) 51:202–6 doi: 10.1038/s41588-018-0312-8.Epub2019.1.14 PMC6365097 30643254

[17] Ye Z Zhang Y Liang Y Lang J Zhang X Zang G. Cervical cancer metastasis and recurrence risk prediction based on deep convolutional neural network. Curr Bioinf (2022) 17:164–73 doi: 10.2174/1574893616666210708143556

[18] Liu J Lan Y Tian G Yang J. A systematic framework for identifying prognostic genes in the tumor microenvironment of colon cancer. Front Oncol (2022) 2:899156. doi: 10.3389/fonc.2022.899156 PMC9161737 35664768

[19] Yin Y Zhang YB Xing LN. Predictive value of tumor mutation burden in immunotherapy for non-small-cell lung cancer. Chin J Multiple Organ Dis Elderly (2018) 9:82–5. doi: 10.11915/j.issn.1671-5403.2018.09.165

[20] Gubin MM Artyomov MN Mardis ER Schreiber RD. Tumor neoantigens: building a framework for personalized cancer immunotherapy. J Clin Invest (2015) 125:3413–21. doi: 10.1172/JCI80008 PMC4588307 26258412

[21] Yarchoan M Hopkins A Jaffee EM. Tumor mutational burden and response rate to PD-1 inhibition. N Engl J Med (2017) 377:2500–1. doi: 10.1056/NEJMc1713444 PMC6549688 29262275

[22] Mansfield AS Ren H Sutor S Sarangi V Nair A Davila J. Contraction of T cell richness in lung cancer brain metastases. Sci Rep (2018) 8:2171. doi: 10.1038/s41598-018-20622-8 29391594 PMC5794798

[23] Yang J Hui Y Zhang Y Zhang M Ji B Tian G. Application of circulating tumor dna as a biomarker for non-small cell lung cancer. Front Oncol (2021) 11:725938. doi: 10.3389/fonc.2021.725938 34422670 PMC8375502

[24] Song Z Chen X Shi Y Huang R Wang W Zhu K. Evaluating the potential of t cell receptor repertoires in predicting the prognosis of resectable non-small cell lung cancers. Mol Ther Methods Clin Dev (2020) 18:73–83. doi: 10.1016/j.omtm.2020.05.020 32995352 PMC7488751

[25] Remon J Besse B Soria JC. Successes and failures: what did we learn from recent first-line treatment immunotherapy trials in non-small cell lung cancer? BMC Med (2017) 15(1):55. doi: 10.1186/s12916-017-0819-3 28285592 PMC5346853

[26] Peters S Creelan B Hellmann M Socinski MA Carbone DP. Abstract CT082: Impact of tumor mutation burden on the efficacy of first-line nivolumab in stage iv or recurrent non-small cell lung cancer: An exploratory analysis of CheckMate 026. Cancer Res (2017) 77(13_supplement):CT082. doi: 10.1158/1538-7445.AM2017-CT082

[27] Matsushita H Vesely MD Koboldt DC Rickert CG Uppaluri R Magrini VJ. Cancer exome analysis reveals a T-cell-dependent mechanism of cancer immunoediting. Nature (2012) 482:400–4. doi: 10.1038/nature10755 PMC3874809 22318521

[28] Rooney MS Shukla SA Wu CJ Getz G Hacohen N. Molecular and genetic properties of tumors associated with local immune cytolytic activity. Cell (2015) 160:48–61. doi: 10.1016/j.cell.2014.12.033 25594174 PMC4856474

[29] Hellmann MD Ciuleanu TE Pluzanski A Lee JS Otterson GA Audigier-Valette C. Nivolumab plus ipilimumab in lung cancer with a high tumor mutational burden. N Engl J Med (2018) 378:2093–104. doi: 10.1056/NEJMoa1801946 PMC7193684 29658845

[30] Ready N Hellmann MD Awad MM Otterson GA Gutierrez M Gainor JF. First-line nivolumab plus ipilimumab in advanced non-small-cell lung cancer (checkmate 568): outcomes by programmed death ligand 1 and tumor mutational burden as biomarkers. J Clin Oncol (2019) 37:992–1000. doi: 10.1200/JCO.18.01042 30785829 PMC6494267

[31] Nadal E Massuti B Dómine M García-Campelo R Cobo M Felip E. Immunotherapy with checkpoint inhibitors in non-small cell lung cancer: insights from long-term survivors. Cancer immunology immunotherapy: CII (2019) 68:341–52. doi: 10.1007/s00262-019-02310-2 PMC11028247 30725206

[32] Vokes EE Ready N Felip E Horn L Burgio MA Antonia SJ. Nivolumab versus docetaxel in previously treated advanced non-small-cell lung cancer (CheckMate 017 and CheckMate 057): 3-year update and outcomes in patients with liver metastases. Ann Oncol (2018) 29:959–65. doi: 10.1093/annonc/mdy041 29408986

[33] Chalmers ZR Connelly CF Fabrizio D Gay L Ali SM Ennis R. Analysis of 100,000 human cancer genomes reveals the landscape of tumor mutational burden. Genome Med (2017) 9:34.doi: 10.1186/s13073-017-0424-2 28420421 PMC5395719

[34] Rizvi NA Hellmann MD Snyder A Kvistborg P Makarov V Havel JJ. Cancer immunology. mutational landscape determines sensitivity to PD-1 blockade in non-small cell lung cancer. Sci (New York N.Y.) (2015) 348:124–8. doi: 10.1126/science.aaa1348 PMC4993154 25765070

